# *Livin/BIRC7* gene expression as a possible diagnostic biomarker for endometrial hyperplasia and carcinoma

**DOI:** 10.1186/s43141-021-00244-w

**Published:** 2021-09-26

**Authors:** Basma K. Elmekkawy, Rasha M. S. Shoaib, Amal K. Seleem, Dalia Shaalan, Entsar A. Saad

**Affiliations:** 1 Chemistry Department, Faculty of Science, Damietta University, Mobark street, New-Damietta, Damietta, 34517 Egypt; 2 Department of Medical Biochemistry and Molecular Biology, Faculty of Medicine, Mansoura University, Mansoura, Egypt

**Keywords:** Endometrial hyperplasia, Endometrial carcinoma, *Livin*, *BIRC7*, Gene expression

## Abstract

**Background:**

*Livin/BIRC7* is a member of the inhibitors of apoptosis proteins family which are implicated in development of cancer through the inhibition of apoptosis process. This case-control study was intended to investigate *livin/BIRC7* gene expression in endometrial hyperplasia and carcinoma and its correlation to some oxidative stress markers in addition to its possible diagnostic performance.

**Methods:**

This study included 90 participants [30 endometrial hyperplasia patients, 30 endometrial carcinoma patients, and 30 healthy controls]. *Livin/BIRC7* gene expression was analyzed using quantitative reverse transcriptase-polymerase chain reaction (RT-PCR). Serum catalase activity was assessed by enzyme-linked immunosorbent assay (ELISA) and malondialdehyde level was measured by the colorimetric method.

**Results:**

*Livin/BIRC7* gene expression was significantly (*p* < 0.001) higher in endometrial carcinoma from patients with endometrial hyperplasia when compared to controls. A positive correlation was found between *livin/BIRC7* expression and serum catalase activity and malondialdehyde level in endometrial hyperplasia and carcinoma. The detection of *livin/BIRC7* in endometrial carcinoma has excellent sensitivity and specificity.

**Conclusions:**

*Livin/BIRC7* was overexpressed in endometrial carcinoma with excellent power to differentiate endometrial carcinoma from endometrial hyperplasia or healthy subjects, suggesting that it might be a useful molecular marker for endometrial carcinoma diagnosis.

## Background

Endometrial hyperplasia is an abnormal proliferation of glands and stroma of the endometrium highlighted with the existence of cytologic and tissue architectural amendments [1]. Endometrial hyperplasia, if not cured, has the tendency to progress into endometrial carcinoma [2]. During carcinogenesis and as a central step for cancer development, malignant cells inactivate apoptosis. They neglect apoptosis signals, which regularly direct cells to stop reproduction or to start cell apoptosis to dispose of the abnormal cancerous cells. This inactivation of apoptotic signals may be a significant subscriber to therapy resistance in several neoplasms [3].

Among the most common cancers worldwide, the endometrial cancer is the 15th and it is the 6th among women cancers with more than 380,000 new diagnosed cases and about 90,000 deaths in 2018. The numbers are rising yearly, with the highest incidence in Canada and the USA [4]. However, in low-income and middle-income countries, the fatality/incidence rate is still high [5]. Extended unlimited estrogen exposure, age, obesity, diabetes, race, nulliparity, infertility, early age of menarche, family history, late age of menopause, and tamoxifen drug are the important threats for the development of endometrial cancer [6]. The percent of 5-year survival in endometrial cancer, without spread to another place, is between 74 and 91 [7]. A great number of the cases are diagnosed at the early stage (I or II) and showed a better prognosis and the possibility of no recurrence after surgery and adjuvant radiotherapy. Nevertheless, from 10 to 15% recurs. Between 80 and 90% of recurrences occur within 3 years [8]. Localized recurrences, which mostly appear in the vaginal vault, are more likely to develop in the early-stage patients treated with surgery solely. More distant recurrences are more probable to happen in patients initially cured by multimodality treatment [https://www.cancertherapyadvisor.com/home/decision-support-in-medicine/obstetrics-and-gynecology/endometrial-chemotherapy-treatment-for-recurrence/]. Many unanswered research questions remain concerning controlling this disease ranging from treatment toxicity, diagnostic procedures to adjuvant therapy [4]. There are no definite lab analyses for the diagnosis of endometrial cancer. The recommended ways are either transvaginal ultrasonography or endometrial biopsy. Therefore, precise diagnosis requires an endometrial tissue sample [9]. Consequently, there is a great responsibility from researchers to seek new biomarkers, which can serve as predictors and/or diagnostics for endometrial carcinoma.

*Baculoviral IAP repeat-containing 7* (*BIRC7*), called *livin* and *livin inhibitor-of-apoptosis* as well, is one of the family members of the inhibitor of apoptosis protein (IAP) and was acknowledged to play a role in the inhibition of cell death prompted by apoptosis and adjustment of cell proliferation and cell cycle [10]. The livin/BIRC7 protein is composed of 298 amino acid residues long with a molecular weight of about 32798 Da, enclosing three PDB structures, seven beta-strands, six turns, and nine helix domains [Data source: Uniprot database (Q96CA5)]. BIRC7 contains a single domain of Baculovirus IAP-Repeat (BIR) comprising approximately 70 amino acids and a zinc-binding RING-domain. BIRC7-BIR is globular in structure stabilized by four α-helices and an anti-parallel sheet of three filaments, in addition to residues that give the hydrophobic nucleus. Additionally, the BIRC7-RING domain is typically distinct by seven cysteines and one histidine which able to coordinate two zinc atoms in its carboxy-terminus (atlasgeneticsoncology.org). The BIR domain is required for inhibitory activity and reacts with caspases or with APAF1 which together with cytochrome C mediates caspase 9 activation, while the zinc-binding RING-domain sometimes rises antiapoptotic activity but does not inhibit apoptosis alone [11].

The human BIRC7 is encoded by *BIRC7* (OMIM#: 605737) gene that positioned on chromosome 20q13.33 and spanned nearly 4613 bases. The *BIRC7* gene consists of seven exons with six introns [12]. Based on the genomic structure of the *livin/BIRC7* gene, it has consisted of three splice variants [BIRC7-201, BIRC7-202, BIRC7-203]. The protein-protein interaction networks implicated that livin/BIRC7 protein has vital functions in the apoptotic process, regulation of catalytic activity, enzyme inhibitor activity, JNK cascade, and signal transduction [Data source: STRING database, version 11.0]. The gene-gene interaction networks indicated that the *livin/BIRC7* gene has crucial functions in the execution phase of apoptosis, endopeptidase inhibitor activity, negative regulation of hydrolase activity, and apoptotic DNA fragmentation [Data source: GeneMANIA database]. The subcellular localizations of the livin/BIRC7 protein indicated that it is detected with abundance in the nucleus, cytoplasm, and Golgi apparatus [Data source: Cellular compartment database].

In general, as most IAPs, *BIRC7* expression was not noticeable in normal differentiated adult tissues, except placenta, spleen, lymph nodes, and developing embryonic tissues [13]. On the other hand, *BIRC7* overexpression has been reported in several cancer types, in which it was associated with malignancy and chemoresistance as in adrenocortical tumors [14], prostate cancer [15], papillary thyroid carcinoma [16], bladder cancer [17], colorectal cancer [18], renal carcinoma [19], hepatocellular carcinoma [20], lung cancer [21], and acute lymphoblastic leukemia [22].

No data were identified concerning the diagnostic or prognostic impacts of *livin/BIRC7* gene expression in endometrial hyperplasia and/or endometrial carcinoma. Thus, our team was motivated to explore the *livin/BIRC7* gene expression in both endometrial hyperplasia and carcinoma, in relation to some oxidative stress markers, and its diagnostic performance to help in developing better treatment strategies.

## Methods

### Study population

This preliminary case-control study included 90 women divided into 3 groups: 30 women with endometrial hyperplasia (group I), 30 women with endometrial carcinoma (group II), and 30 healthy controls (group III). They were diagnosed and recruited between November 2017 and December 2019 from the Obstetrics and Gynecology Clinic of Mansoura University Hospital, Mansoura, Egypt. Only endometrial hyperplasia cases diagnosed with simple endometrial hyperplasia without atypia were included. They were with a mean age of 49.73 years. Only endometrial carcinoma cases diagnosed with endometrial carcinoma of stage I were included. They were with a mean age of 52.16 years. Patients were at pre-menopause and post-menopause stages. Healthy controls were randomly selected of matched sex and mean age (women with a mean age of 51.83 years). Women with endometrial hyperplasia of other types, with endometrial carcinoma of higher stages, with chronic non-specific endometritis and fibroid were excluded. Clinical characteristics of the study groups are listed in Table 1. Written informed consents were gotten from all study contributors with a declaration of data privacy.

**Table 1:** Demographic and biochemical data of the studied groups

	Endometrial hyperplasia (*n* = 30)	Endometrial carcinoma (*n* = 30)	Control (*n* = 30)
Age (years)	49.73 ± 1.07	52.16 ± 1.08	51.83 ± 1.17
*p* value	0.12^*^	0.79^$^	0.19^!^
Bodyweight (kg)	93.47 ± 2.71	77.67 ± 2.12	84.70 ± 2.58
*p* value	< 0.001^*^	0.06^$^	**0.02**^!^
Estrogen (pg/mL)	30.87 ± 2.34	26.80 ± 1.75	29.67 ± 2.06
*p* value	0.17^*^	0.33^$^	0.68^!^
Catalase (mU/mL)	0.18 ± 0.01	0.21 ± 0.02	0.17 ± 0.01
*p* value	0.093^*^	**0.027**^$^	0.57^!^
Malondialdehyde (μmol/L)	6.51 ± 0.28	9.09 ± 0.36	3.17 ± 0.21
*p* value	< 0.001^*^	**< 0.001**^$^	**< 0.001**^!^

*M ± SE*: mean ± standard error; pairwise comparison between each 2 groups was done using post hoc test

^*^Comparison between endometrial hyperplasia and endometrial carcinoma

^$^Comparison between endometrial carcinoma and control

^!^Comparison between control and endometrial hyperplasia

### Samples collection

Five milliliters of peripheral venous blood was received from all study contributors using sterile venipuncture and plastic disposable syringes. Each sample was portioned into two tubes; 3 mL blood without anticoagulant and centrifuged for 15 min at 5000 rpm to get serum for assessment of estrogen level, catalase activity, and malondialdehyde level. Two milliliters of blood with ethylene diamine tetraacetic acid was used for RNA extraction.

### Estimation of estrogen hormone level

The estrogen hormone level was determined by the quantitative competitive enzyme-linked immunosorbent assay (ELISA) kit (Sigma-Aldrich, SE120049, USA) following the kit instructions.

### Estimation of malondialdehyde (MDA) level

The serum MDA level was measured by the colorimetric method kit (Elabscience, E-BC-K025-M, USA) following the manufacturer instructions.

### Estimation of catalase enzyme activity

The serum catalase activity was assessed according to the manufacturer instructions of the colorimetric method kit (Abcam, ab83464, USA).

### *Livin/BIRC7* gene expression by RT-PCR

Total RNA isolation was done using a Tri-FastTM reagent kit (PeqLab Biotechnologie GmbH, Germany) according to the Chomczynski method [23]. Reverse transcriptase-polymerase chain reaction (RT-PCR) was done using the QIAGEN One-step RT-PCR kit (QIAGEN, Valencia, USA) according to McPherson and Moller method [24]. Each sample was analyzed in duplicate. Quantitative RT-PCR is then performed as follows: *Livin/BIRC7* gene-specific primers (Biolegio BV, Nijmegen, Netherlands): Forward: (5′-d [ATG GGC TCT GAG GAG TTG CGT C]-3). Reverse: (5′-d [CAT AGC AGA AGA AGC CC TCA CCT TG]-3′). Internal control (GAPDH) gene-specific primers: Forward: (5′-d [CGG AGT CAA CGG ATT TGG TCG TAT]-3′). Reverse: (5′-d [AGC CTT CTC CAT GGT GGT GAA GAC]-3′). The reaction mixture was performed in a final volume of 50 μL containing 40 μL of master mix (20 μL RNase free water, 10 μL of 5× RT-PCR buffer, 2 μL dNTP mix which is 10 mM of each dNTP, 3 μL sense primer, 3 μL antisense primer, and 2 μL RT-PCR enzyme mix) and 10 μL of each template RNA. Reactions were performed using TECHNE TC-312 thermal Cycler PCR. Amplification steps involved 15 min of initial PCR activation at 95 °C, then 35 cycles of 1 min of denaturation at 94 °C, 1 min of annealing at 58 °C, and 1 min of extension at 72 °C. A final extension was done at 72° for 10 min. PCR products were electrophoresed on 2% agarose gel and visualized on the UV transilluminator. The obtained bands were digitally photographed under fixed conditions (the light, the distance, and the zoom). Photos analysis was carried out via using the scion image® release Alpha 4.0.3.2 software for windows® which accomplishes detection of bands and conversion to peaks. Areas under each peak were calculated in square pixels and used in quantification. *Livin/BIRC7* gene expression was determined by calculating the ratio between the square pixel value of the *Livin/BIRC7* gene in relation to the GAPDH gene.

### Statistical analysis

The data processing, manipulation, and organization were presented utilizing the IBM SPSS statistics program, version 26.0 (Thousand Oaks, CA, USA). The normal distribution of the study data was assessed using the Kolmogorov–Smirnov test. The continuous variables were processed as mean ± standard error (SE) using the one-way ANOVA test. Pairwise comparison between every 2 groups was done using post hoc test (Dunn’s test) for multiple comparisons test. Correlations between the clinical variables were analyzed using the Pearson correlation test. The receiver operating characteristic (ROC) curve was plotted to determine the cut-off point. *p* ≤ 0.05 was considered significant. The boxplot was carried out by the R programming Language (version 4.0.3) with RStudio open source (version 1.4.1103).

## Results

### Demographic and biochemical data of the studied groups

The demographic and biochemical data of all studied groups are presented in Table 1. This study involved 90 women divided into three groups: group 1—30 women with endometrial hyperplasia with a mean age ± standard error (SE) was 49.73 ± 1.07 years; group 2—30 women with endometrial carcinoma with a mean age ± SE was 52.16 ± 1.08 years, and group III—30 healthy controls with a mean age ± SE was 51.83 ± 1.30 years. The groups were well balanced in terms of age and estrogen level. The mean weight was higher in patients with endometrial hyperplasia compared to endometrial carcinoma patients and controls (*p* < 0.001 and *p* = 0.02; respectively). Catalase activities of endometrial carcinoma cases were higher than those of the control cases (*p* = 0.027). Additionally, the MDA levels were significantly elevated among endometrial carcinoma in comparison to endometrial hyperplasia patients and controls (*p* value < 0.001) (Table 1).

### *Livin/BIRC7* expression in endometrial hyperplasia, endometrial carcinoma, and healthy controls

The expression of *Livin/BIRC7* as shown in Table 2 was elevated among patients with endometrial hyperplasia and endometrial carcinoma in comparison to controls (*p* < 0.001). Besides, the *livin/BIRC7* expression was higher in patients with endometrial carcinoma (1.59 ± 0.11) than that in endometrial hyperplasia patients (0.68 ± 0.06) (*p* < 0.001).

**Table 2:** *Livin/BIRC7* expression level in the studied groups

Studied groups	*Livin/BIRC7* expression	*p* value
Endometrial hyperplasia (*n* = 30)	0.68 ± 0.06	< 0.001*
Endometrial carcinoma (*n* = 30)	1.59 ± 0.11	**< 0.001**^$^
Control (*n* = 30)	0.17 ± 0.03	**< 0.001**^**!**^

*M ± SE*: mean ± standard error

^*^Comparison between endometrial hyperplasia and endometrial carcinoma

^$^Comparison between endometrial carcinoma and control

^!^Comparison between control and endometrial hyperplasia

### Bio-statistical correlations between the studied parameters

*Livin/BIRC7* expression and the bodyweight of studied populations was negatively correlated (*r* = −0.24). On the contrary, significant positive correlations were observed between the *livin/BIRC7* expression, catalase (*r* = 0.22) and MDA (*r* = 0.66). Moreover, the activity of catalase correlated positively with MDA level (*r* = 0.23) as demonstrated in Table 3. No significant correlations were found between estrogen level and any of the studied parameters.

**Table 3:** Pearson’s correlation analysis among all the studied parameters

Parameters	***Livin/BIRC7*** expression		Age		Bodyweight		Estrogen		Catalase	
	***r***	***p***	***r***	***p***	***r***	***p***	***r***	***p***	***r***	***p***
***Livin/BIRC7*** **expression**										
**Age**	0.06	0.56								
**Bodyweight**	−0.24	0.03*	−0.003	0.98						
**Estrogen**	−0.07	0.49	−0.003	0.98	0.06	0.58				
**Catalase**	0.22	0.04*	−0.04	0.74	−0.06	0.56	−0.14	0.18		
**Malondialdehyde**	0.66	< 0.001*	0.04	0.68	−0.19	0.07	−0.09	0.35	0.23	0.03*

*r*: parametric correlation (Pearson *r*)

^*: ^*p* ≤ 0.05 is significant

### Diagnostic performance

The ROC curve analysis was applied to assess the power of *livin/BIRC7* expression, catalase activity, and MDA levels in discriminating between endometrial carcinoma, hyperplasia, and normal endometria. As regard to predicting endometrial hyperplasia (Table 4), *livin/BIRC7* expression and MDA were significant (*p* < 0.001), AUC = 0.95 and 0.97 respectively compared to 0.53 for catalase (*p* = 0.66). The MDA had better sensitivity (100%) whereas *livin/BIRC7* expression had better specificity (90%). Similarly, *livin/BIRC7* expression and MDA were significant (*p* < 0.001), AUC = 0.998 and 0.991 respectively compared to 0.613 for catalase (*p* = 0.13) in predicting endometrial carcinoma (Table 5). The *livin/BIRC7* expression had better sensitivity (100%) while MDA had better specificity (100%). In differentiating endometrial carcinoma from endometrial hyperplasia (Table 6), *livin/BIRC7* expression was the most powerful with AUC = 0.94 compared to 0.87 and 0.59 for MDA and catalase respectively. The *livin/BIRC7* expression had better sensitivity (93.3%).

**Table 4:** Diagnostic performance of *livin/BIRC7* expression, catalase, and malondialdehyde (MDA) for prediction of endometrial hyperplasia

	***Livin/BIRC7*** expression	Catalase	MDA
**Cutoff**	> 0.30	> 0.14	> 4.12
**AUC**	0.950	0.530	0.970
**Sensitivity %**	96.7%	70.0%	100%
**Specificity %**	90.0%	36.7%	83.3%
**Accuracy**	86%	7%	83%
**PPV %**	90.6%	52.5%	85.7%
**NPV %**	96.4%	55.0%	100.0%
**95% CI**	0.87-0.99	0.39-0.66	0.89-0.99
***p*** **value**	**< 0.001**	0.66	**< 0.001**

*AUC*: area under the curve, *PPV*: positive predictive value, *NPV*: negative predictive value, *CI*: confidence interval

**Table 5:** Diagnostic performance of *Livin/BIRC7* expression, catalase, and malondialdehyde (MDA) for prediction of endometrial carcinoma

	***Livin/BIRC7*** expression	Catalase	MDA
**Cutoff**	> 0.60	> 0.22	> 5.21
**AUC**	0.998	0.613	0.991
**Sensitivity %**	100%	36.7%	96.7%
**Specificity %**	96.7%	93.3%	100%
**Accuracy**	97%	30%	97%
**PPV %**	96.8%	84.6%	100%
**NPV %**	100%	59.6%	96.8%
**95% CI**	0.94-1.00	0.48-0.74	0.92-1.00
***p*** **value**	**< 0.001**	0.13	**< 0.001**

*AUC*: area under the curve, *PPV*: positive predictive value, *NPV*: negative predictive value, *CI*: confidence interval

**Table 6:** Diagnostic performance of *Livin/BIRC7* expression, catalase, and malondialdehyde (MDA) for differentiation between endometrial carcinoma and endometrial hyperplasia

	***Livin/BIRC7*** expression	Catalase	MDA
**Cutoff**	> 0.90	> 0.22	> 7.30
**AUC**	0.940	0.590	0.870
**Sensitivity %**	93.3%	36.7%	90.0%
**Specificity %**	86.7%	86.7%	83.3%
**Accuracy**	80%	23%	73%
**PPV %**	87.5%	73.3%	84.4%
**NPV %**	92.9%	57.8%	89.3%
**95% CI**	0.84-0.98	0.45-0.71	0.76-0.94
***p*** **value**	**< 0.001**	0.24	**< 0.001**

*AUC*: area under the curve, *PPV*: positive predictive value, *NPV*: negative predictive value, *CI*: confidence interval

## Discussion

Globally, endometrial cancer is the 6th utmost common cancer in females and the utmost common malignancy of the female genital area [25]. Predictive, diagnostic, and prognostic biomarkers were identified for several other cancers [26–28]. The present study was the pioneer to investigate the role of *livin/BIRC7* gene expression in endometrial hyperplasia and carcinoma.

A vital mechanism involved in tumorigenesis is the inhibition of apoptosis [29]. There have been accumulated evidence that overexpression of *BIRC7* in cancers has been connected to chemo- and radio-resistance, relapse, and poor survival [30]. The reported data that in normal tissues *Livin/BIRC7* is ordinarily not detected [31], but overexpressed in malignant tissues was confirmed by our results which demonstrated that *livin/BIRC7* gene is over-expressed in our patient groups 1 and 2. The expression was significantly higher in carcinoma than hyperplasia suggesting that the more the severity of the disease is the greater the level of expression. The results also recommended *livin/BIRC7* gene over-expression as a true risk factor for endometrial carcinoma. Several mechanisms have been established by which *livin/BIRC7* over-expression enhances malignancies [32]. Among these, the antiapoptotic activity of *livin/BIRC7* is mediated through inhibition of caspase-3, -7, and -9, as well as by its E3 ubiquitin-ligase-like activity which stimulates Smac/DIABLO degradation which plays a crucial role in the activation of caspases [33]. It was also found that there were two similar splicings of *livin*, *livin-α*, and *livin-β* that could be distinguished by the presence of 18 amino acids positioned at the interval of the BIR and RING domains in the α-isoform. Although they are similar in structure, the different isoforms show different anti-apoptotic features [34] and consequently differ in their tumorigenic manner.

According to our findings, the bodyweight was significantly higher in patients with endometrial hyperplasia confirming high bodyweight as a risk factor for endometrial hyperplasia. However, in endometrial carcinoma, bodyweight showed a tendency to decline. This was ascertained further in our work by the significant negative correlation found between bodyweight and the *livin/BIRC7* gene expression. Bodyweight decline in carcinoma may be due to the predominance of the catabolic status at the expense of the anabolic status and the change to anaerobic metabolism due to oxygen lack leading to energy waste via the Cori cycle. As the *livin/BIRC7* gene expression was significantly upregulated and was further higher in endometrial carcinoma patients than in endometrial hyperplasia cases. Similar behavior was observed for MDA as well; it was significantly elevated in endometrial hyperplasia and recorded further elevation associated with endometrial carcinoma confirming the great association in lipid peroxidation with disease development and progression. High levels of reactive species have anti-apoptotic effects in tumor cells that appear as a result of redox-sensitive transcription factors activation [35] resulting in triggering cellular proliferation, cell migration, and invasion, all of which participate in carcinogenesis [36]. Surprisingly, in the current study, the antioxidant enzyme catalase was significantly highly activated in endometrial carcinoma cases compared to controls while it showed no difference between endometrial hyperplasia cases and controls in its activity. A possible explanation could be that in response to acute oxidative stress of endometrial hyperplasia, antioxidants including catalase may be expended to prevent the oxidative-dependent damage. This is in the same line with Ota et al. [37]. Nevertheless, the elevation in catalase activity in endometrial carcinoma may be one of the adaptive mechanisms exerted by the host body against excessive produced reactive species by the tumor cells. As the prolonged oxidative stress raises the production of reactive species and assumed to encourage antioxidants more release. Interestingly, this may be confirmed in light of the observed significant positive correlation between catalase activity and each of MDA and *livin/BIRC7* gene expression. Therefore, our findings in the current study clearly propose that *livin/BIRC7* over-expression and oxidative stress status can be affected by/and could be associated with the endometrial disease severity, chronicity, and progression. It has been reported that endometrial hyperplasia and carcinoma were associated with increased lipid peroxidation and altered antioxidant enzymes activities [38] and it was suggested [39] that lipid peroxidation elevation appears to be a consequence of the disease rather than its etiology.

In the current study, using ROC curve analysis, we found that *livin/BIRC7* expression and MDA level but not the catalase could be used as additional dependable parameters in the diagnosis of the suspected cases with endometrial carcinoma and endometrial hyperplasia. Both *livin/BIRC7* and MDA are excellent to distinguish endometrial hyperplasia cases from control subjects. However, MDA is better regarding AUC value (0.97 with accuracy of 83% vs. 0.95 with accuracy of 86% for *livin*). Also, they are excellent for differentiation of endometrial carcinoma cases from controls; however, *livin/BIRC7* is more powerful than MDA regarding AUC value (0.998 vs. 0.991) with the same accuracy (97%) for the two. Regarding differentiation of endometrial carcinoma from endometrial hyperplasia, the performance of both still great but *livin/BIRC7* is superior regarding AUC value and accuracy (0.94 with accuracy of 80% for *livin/BIRC7* vs. 0.87 with accuracy of 73% for MDA).

Some limitations were raised upon the establishment of the present work that being a single institutional study with a small sample size which might affect sensitivity of the investigated gene expression as a predictive and prognostic biomarker. Moreover, apoptotic markers in endometrial carcinoma should be studied in correlation with *livin/BIRC7* gene expression.

## Conclusions

In conclusion, the current study demonstrated that *livin/BIRC7* and lipid peroxidation were over-expressed/elevated in endometrial carcinoma compared to endometrial hyperplasia. We may suggest *livin/BIRC7* expression in endometrial hyperplasia as a marker with a good diagnostic accuracy (area under ROC curve, 0.950), sensitivity (96.7%), and specificity (90.0%). While in endometrium carcinoma, we detected gene expression of high sensitivity and specificity, 100% and 96.7% respectively. Therefore, our findings provided evidence that the detection of *livin/BIRC7* may be used as a reliable diagnostic biomolecular technique for endometrial carcinoma.

## Data Availability

Data is not available due to [ethical/legal/commercial] restrictions: Due to the nature of this research, participants of this study did not agree for their data to be shared publicly, so supporting data is not available.

## Abbreviations

RT-PCR: Reverse transcriptase-polymerase chain reaction
ELISA: Enzyme-linked immunosorbent assay
*BIRC7*: *Baculoviral IAP repeat-containing 7*
IAP: Inhibitor of apoptosis protein
BIR: Baculovirus IAP-repeat
MDA: Malondialdehyde
ROC curve: Receiver operating characteristic curve
SE: Standard error
AUC: Area under the curve
