# Correction to: *Livin/BIRC7* gene expression as a possible diagnostic biomarker for endometrial hyperplasia and carcinoma

**DOI:** 10.1186/s43141-022-00308-5

**Published:** 2022-02-11

**Authors:** Basma K. Elmekkawy, Rasha M. S. Shoaib, Amal K. Seleem, Dalia Shaalan, Entsar A. Saad

**Affiliations:** 1 Chemistry Department, Faculty of Science, Damietta University, Mobark street, New-Damietta, Damietta, 34517 Egypt; 2 Department of Medical Biochemistry and Molecular Biology, Faculty of Medicine, Mansoura University, Mansoura, Egypt

**Correction to: J Genet Eng Biotechnol 19, 1-8 (2021)**

**https://doi.org/10.1186/s43141-021-00244-w**

Following publication of the original article [1], the authors identified an error in the author name of Dalia Shaalan.

The incorrect author name is:

Dalia Shalaan

The correct author name is:

Dalia Shaalan

The author group has been updated above and the original article [1] has been corrected.
